# Adipose stem cells in reparative goat mastitis mammary gland

**DOI:** 10.1371/journal.pone.0223751

**Published:** 2019-10-22

**Authors:** Clautina R. M. Costa, Matheus L. T. Feitosa, Andressa R. Rocha, Dayseanny O. Bezerra, Yulla K. C. Leite, Napoleão M. Argolo Neto, Huanna W. S. Rodrigues, Antônio Sousa Júnior, Adalberto S. Silva, José L. R. Sarmento, Lucilene S. Silva, Maria A. M. Carvalho

**Affiliations:** 1 Integrated Nucleus of Morphology and Stem Cell Research (NUPCelt), Federal University of Piauí (UFPI), Teresina, Piauí, Brazil; 2 Technical College of Teresina, Federal University of Piauí, Teresina, Brazil; 3 Biology Department, Federal University of Piauí, Teresina, Piauí, Brazil; University of Illinois, UNITED STATES

## Abstract

Mesenchymal stem cells have been widely used in the treatment of various chronic diseases. The objective of this survey was to evaluate the therapeutic and regenerative potential of stem cells from adipose tissue (ASCs) in the milk production recovery repair of tissue injury in mastitis goats treated with antimicrobial agents prior to cell therapy. After the diagnosis of mastitis and treatment with gentamicin, eight lactating goats were selected for cellular and subsequent therapy, physical-chemical analysis of milk, ultrasonographic and histopathological examinations. The ASCs were taken from the subcutaneous fat of a young goat cultivated *in vitro*, marked with Qdots-655 and injected in the left mammary gland, being the right mammary gland used as the control. After 30 days the ultrasonographic and histopathological analyzes were repeated and, in the first lactation period, the physical-chemical analysis of the milk was reapeated. Before the cellular therapy, the physical-chemical quality of the milk was compromised and the ultrasonographic and histopathological analysis revealed a chronic inflammatory process and fibrous tissue. The marking of the ASCs with Qdots enabled the tracking, by fluorescence microscopy (BX41-OLYMPUS), in the mammary tissue. In the ASCs therapy, cultures showed high cellularity and characteristics favorable to preclinical studies; with the therapy the physical-chemical parameters of the milk, fat, protein, temperature and pH showed significant differences among the groups; five animals treated with ASCs reconstituted the functionality of the gland and the connective tissue reduced in quantity and inflammatory infiltrate cells. ASCs have potential for the possible regeneration of fibrous mastitis lesions in the mammary gland, however, it would be necessary to increase injection time for the histopathological analysis, since the reconstitution of the glandular acini within the assessed period was not finalized. ASCs can be used to reestablish milk production in goat with chronic mastitis repair mammary lesions, with potential to be a promising clinical alternative for animal rehabilitation for productivity.

## Introduction

Cell therapy is an alternative for the treatment of various chronic and degenerative diseases. Stem cells can reverse pathological condition of various diseases in almost all body tissues [1, 2].

Mastitis is a disease of the mammary gland responsible for irreversible changes in the glandular tissue, and there are no effective drugs or therapy methods that can completely lessen the characteristics of the lesions, which directly affect milk production, causing great harm to the producers [3]. As a result of the inflammatory process, a large proportion of the mammary tissue is replaced by cicatricial connective tissue [4, 5].

These cells represent a lineage of mesenchysome cells, with extensive proliferative capacity, in addition it differs itself in various types of cells [5, 6, 7, 8, 9, 10, 11]. In vitro culture is characterized by cell adhesion to the plastic, colony forming capacity, low immunogenic phenotype and costimulatory molecules [12, 13].

ASCs have a great potential for angiogenesis [14], so if injected into the mammary gland of goats with chronic mastitis it may restore its functionality, since it is a richly vascularized organ. In the case of therapies, ASCs have other favorable characteristics, since they have a regenerative stimulating effect, are abundant, accessible, easy to obtain and multidirectional differentiation potential, they express on the surface high levels of markers, among which the most frequently reported are CD34, CD90, CD146, CD105 AND CD73 [15, 16, 17]. The ASCs showed good results in repair therapy of sciatic nerve injury applied in vivo in a study conducted by DiMarino et al. [18].

Obtaining the subcutaneous adipose tissue is pointed out in the literature [19] as an easy technique, in addition, the number of cells available in these depots are very and thus the best alternative for clinical application [20, 21].

Research with ASCs has focused on its positive potentials, especially regarding the healing process, such as repair of rabbit tendon [21], vocal fold in a canine model [10], neurodegenerative conditions in murine model [22]. In goats’ mammary glands, up to now, no studies were found with the use of these cells.

The characteristics of multipotentiality and immunomodulation of mesenchymal stem cells, associated with previous reports of their efficacy in repairing somatic tissues, make it a promising alternative for the treatment of caprine mastitis. However, this hypothesis still lacks validation. For This reason, the objective of this study was to evaluate the therapeutic contribution of ASCS in the morpho-functional reestablishment of milk production of the goats’ mammary glands with chronic spontaneous mastitis.

## Materials and methods

### Animals and ethical aspects

All the procedures of this study were authorized by the Animal Experimentation Ethics Committee of the Federal University of Piauí (n° 037/2012), following the rules of the National College of Animal Experimentation Control (S1 File). The goats with spontaneous mastitis were kept in the goat breeding sector, fed with native pasture and elephant grass (*Pennisetum Purpureum Schum*) intercropped with grasses, in addition to commercial ration pelleted with 18% crude protein and *ad libitum* water. After the experiment, all the animals recovered from the clinical mastitis and were reintegrated into the herd.

### Selection of animals

The animals are from the goat sector of the Federal University of Piauí, Department of Animal Science. Initially 30 goats (*Capra Hircus*) of the breed Anglonubian were selected (same period of lactation), a phase that occurred during the rainy season (February to May). The selection followed the steps: same lactation period, between 4 and 6-year-old, physiological parameters (temperature—39°, heartbeat—72, respiratory movements—30 per min and ruminal movements—3 in 2 min), *California Mastitis Test* positive–CMT [23] and somatic cell count (SCC) positive—>1x10^6^ cells mL^-1^ (Table 1). For SCC, an electronic method was used (DeLaval Cell Count^®^—Direct Cell Countern Delaval), 0.6 mL of milk was aspirated with a disposable cassette, and analyzed in reading equipment. This emits a beam of light that crosses the cassette and in 45 seconds, the individual cell count is performed in SCC/μL.

**Table 1:** Criteria for selection of animals with chronic mastitis.

Order	Selection Criteria	Number of animals with chronic mastitis	Right udder	Left udder
1st ↓	Goats with the same lactation period	30	-	-
2nd ↓	Between 4 and 6-year-old	28	-	-
3rd ↓	Physiological Parameters	28	-	-
4th ↓	Positive CMT* (2+,3+)	10	10	10
5th ↓	SCC >1x10^6^cels mL^-1^	8	8	8

*scores CMT: 1+ (weak positive), 2+ (positive reaction), 3+ (strong positive)

The selected animals were also submitted to examinations to clinic characterization of the mastitis, where the two halves of the mammary gland were analyzed separately for each test suggested totaling 16 samples: microbial cultivation and isolation—conventional techniques of identification of the microbial genus [24]; tests of antimicrobial susceptibility–Mueller-Hinton agar [25]; and the physicochemical analysis of the milk before the suggested treatment—ultrasonic method (Ekomilk Total ^®^ Ultrasonic Milk Testing Device. Eon Trading LLC, Bulgaria), in this test, fat, milk solid-not-fat (MSNF), density, protein, temperature, lactation, electrical conductivity and pH were evaluated to check milk quality; ultrasonographic test, with linear transducer 3.5 to 7 Mhz (Chison Medical Imagine, Model D600VET digital. Series D6V: 10008068) and histopathologic analysis from biopsies.

For the physicochemical analysis of the milk by the ultrasonic method, the udder was sanitized (water and liquid soap and dried with paper towel) and the first three jets of milk were rejected, 200 mL were milked separately from each udder in sterile and identified vials. In the Laboratory of milk analysis from the Núcleo de Estudos, Pesquisas e Processamento de Alimentos (Nucleus of Studies, Research and Food Processing)–NUEPPA/UFPI, each sample was evaluated in triplicate.

In the evaluation by ultrasound examination, a high-resolution portable device was used with a linear transducer 3.5 to 7 Mhz (Chison Medical Imagine, Model D600VET digital. series D6V: 10008068), the images were made with 8cm of depth, 5 MHz of frequency and a 5 MHz probe was used. The animals were kept in a vertical position in the containment trunk, subjected to sanitization of the udder with soap and water and dried with paper towels. After applying the colorless conductive gel the transducer was positioned and moved on the surface of the skin allowing the formation of ultrasonograms in the upper and lower part of each mammary gland.

In order to perform the mammary biopsy procedure, aimming to characterize the mammary tissue before and after treatment, the animals were sedated with ketamine hydrochloride (5 mg/Kg) and antisepsis routine was performed with alcohol 70% followed by Iodine Povidone and subcutaneous infiltration of lidocaine hydrochloride with vasoconstrictor at a dose of 4 mg/Kg was performed in the two biopsy points. Two excisions of 1 cm^2^ were performed, one in each gland, lateral and medial regions, divulsioned with blunt-tipped scissors *Metzembaum*, until visualization of the parenchyma of the mammary tissue and removed by excision, two fragments of three grams, to the fixation in Bouin and freezing, respectively.

The fragments fixed in a solution of Bouin were sent to the Laboratório de Morfologia do Núcleo Integrado de Morfologia e Pesquisa com Células- Tronco (Morphology Laboratory of the Integrated Nucleus of Morphology and Research with Stem Cells (NUPCelt) for routine histopathologic processing and reading of the slides. After paraffin inclusion, 5-μm cuts were made and slides were prepared for coloring with Hematoxylin-Eosin and Masson’s Trichrome for detection of infiltrate of inflammatory cells and fibrous elements and posterior evaluation in optical microscope (Nikon Eclipse E200).

The analysis of the histopathologic slides was performed semi-quantitative, where the intensity of the identified lesions were measured, considering morphologic representative parameters. To do so, it was stablished established scores for stromal fibrosis, based on the density of intralobular and interlobular collagen fibers, such as 0 –absence, 1-mild, 2-moderate, and 3-severe.

For analysis of the infiltrate of inflammatory cells we used methodology of Camperio et al. [26], 0- absence of lesions, absence of interstitial and/or alveolar inflammatory cell infiltrate and undamaged tissue, 1- focal to multifocal, slight interstitial and/or alveolar inflammatory cell infiltrate and undamaged tissue, 2- moderate interstitial and/or multifocal alveolar of inflammatory cells infiltrate and undamaged tissue 3- interstitial and/or diffuse alveolar infiltrate of inflammatory cells and focal areas of tissue injury, 4- interstitial and/or diffuse alveolar infiltrate, severe inflammatory cells and extensive necrotic areas.

The index of epithelial cell proliferation and organization in the mamarium tissue was established by scores, cosidering the celular colonization and density such as, 0 –absence, 1 –mild (neoformation ≤ 25%), 2- moderate (neoformation ≥ 25 ≤ 50%, 3-intense (> 50%). A descriptive analysis of the slides was also performed.

The frozen tissues were analyzed without coloring. The monitoring of ASC marked with nanocrystals (ASC-nac) was performed by fluorescence microscopy (BX41-OLYMPUS).

Diagnostic methods allowed the selection of eight goats with chronic mastitis for g-ASC therapy. Antibiotic therapy for mastitis was performed with gentamicin, 4 mg/Kg daily, for ten days, before treatment with ASCs.

### Isolation and expansion of g-ASCs

The stem cells were obtained from the subcutaneous adipose tissue of young goat (10 months) of the breed Anglonubian, evaluated on weight (40kg), physiological parameters—temperature (39°), heartbeat (72bpm), respiratory movements (30 per min), and ruminal (3 in 2 min) and palpation of the lumbar and sternal region (score 5) [27] for confirmation of the healthiness, transported to the Center of Pre-Clinical Studies (CEPREC) of the Integrated Nucleus of Morphology and Research with Stem Cells (NUPCelt).

It was performed the trichotomy of the sternal (Mediastinal Pectoral region of the 3rd sternebra), local antisepsis with iodinated alcohol and pre anesthesia with ketamine hydrochloride (Dopalen^^®^^, Agribrands. Paulínia, SP, Brazil) at the dose of 8 mg/Kg associated with midazolam (Dormire.^^®^^, Cristália Chemicals and Pharmaceuticals Products, Itapira/SP, Brazil) with 0.5 mg/Kg intramuscularly, and infiltrative subcutaneous anesthesia with 4 mg/Kg of lidocaine hydrochloride (Cristália Chemicals and Pharmaceuticals Products, Itapira/SP, Brazil). It was performed a 1-cm excision of the skin, and divulsed with blunt-tipped scissors up to the subcutaneous adipose tissue and removed a three-gram fragment.

The fat was packaged in Falcon 50 mL tubes (Falcon; BD Pharmingen) with medium DMEM/F12 culture medium (Gibco® Dulbecco's Modified Eagle Medium: Nutrient Mixture F12, Invitrogen, California, USA) and transported to Stem Cell Culture Laboratory (LABCelt).

For the isolation and expansion of g-ASC, the tissue was washed three times with PBS 0.15 M (PBS Gibco^^®^^, Invitrogen, California, USA) containing penicillin (100UI/mL)-streptomycin (10μg/mL) at 4% (Sigma ^®^, St Louis, MO, USA) for elimination of any type of contamination. In Petri dishes with DMEM-F12 (Gibco) culture medium, the fat was fragmented with scalpel and 1 mg/ml of collagenase type I (Sigma, St Louis MO, USA) diluted 1: 3 was added in culture medium without fetal serum, submitted to the incubator at 37° C, 5% CO_2_ for 30 minutes, and one hour in a 37°C water bath shaking the tube every 10 minutes. The suspension was filtered in a 50 mL tube with 100 μm nylon mesh (cell strainer, BD-Biociences, USA). It was centrifuged (Excelsa^®^ Model 280, FANEM, SP, Brazil) twice at 462 g for 10 minutes and the *pellet* resuspended in complete DMEM-F12. Cells were plated in polystyrene culture flasks (Tecno Plastic Products, Switzerland), 25 cm^2^, in the concentration of 2 × 10^6^ cells and maintained in an incubator at 37° C with 5% CO_2_. After 24 hours the culture media was changed. They were kept in culture with successive subcultures every three days until the sixth passage and then frozen. Cell images in culture were visualized under inverted light microscopy (COLEMAN NIB–100^®^).

The evaluation of the growth of g-ASCs was carried out, culturing 1 × 10^5^ Cells/mL in 20 flask of cultivation (25 cm^2^). Every 24 hours, a vial with ASCs was tripsinized and the cells counted in Neubauer chamber (Improved, Labor-Optik, Germany). The way of cultivation of the other vials was changed every three days. The cell count in light inverted microscope (COLEMAN NIB– 100^®^) was performed using the method of exclusion with Trypan Blue 0.4% (Sigma-Aldrich, USA) [28], to determine the quantity and viability of the cells in triplicate. It was used the formula for calculating the cell count (Total cell number x 2 (dilution factor) x 10^4^ (number of quadrants).

### Characterization of g-ASC

For differentiation the culture g-ASCs were detached with Trypsin-EDTA (Invitrogen, Carlsbad, CA, USA) counted and replated (1x10^4^) in a 6-well plate with 2 ml of culture medium, upon reaching confluence of 80% the medium was replaced with commercial media to induce osteogenic, chondrogenic, and adipogenic differentiation (StemPro^®^osteogenesis, StemPro Chondrogenesis and StemPro adipogenesis—Differentiation Kit- Gibco ^™^, Invitrogen, California, USA), changing medium every three days for 21 days. After acquiring morphological characteristics that suggest the expected lineages, they were stained according to the cell type: adipogenic (Oil Red O—Invitrogen Life Science Technologies, Carlsbad, CA, USA), osteogenic (Alizarin Red—Invitrogen Life Science Technologies, Carlsbad, CA, USA), chondrogenic (Alcian Blue—Sigma-Aldrich).

The cells were also characterized according to the presence of mesenchysome stem cell markers (CD90) and absence of hematopoietic stem cell markers (CD14) through the technique of flow cytometry. For the identification of the antigens, the following antibodies were used: CD14 (Anti-CD14 FITC-Sigma, USA, 1:100, Code C7673) and CD90 (Anti-CD90 APC- Abcam Cambridge, USA, 1:100 Code 555596). In addition to a negative control sample only with cells without using antibodies.

The cells in sixth passage were, trypsinized, and after verifying the cellular viability with Trypan Blue, they were counted in Neubauer chamber (Improved, Labor-Optik, Germany) and resuspended in PBS, subsequently 2.5 x 10^5^ cells were distributed in Falcon tubes of 15 mL for each of the markers to be analyzed. In each tube it was placed 1 mL FACS [DBPS] (Dubelco´s Phosphate Buffer Solution) containing 0.1% of BSA, 462g centrifuged for 10 minutes to washing. The cells were incubated with the antibody conjugated for 30 minutes at room temperature. After incubation the cells were washed once with 1 mL FACS buffer for removal of excess antibody. The samples were analysed using a flow rate cytometer (FACScanto® II and BD software FACSDiva Software. Version 6.1.3), obtaining 30.000 events per sample tested. The populations were estimated by the percentage of the cells expressing each one of the markers in compared to the total number of cells acquired using the INFINICYT software (version 5.1). The results were plotted in the form of histogram.

### Marking, transplantation and tracking of g-ASCs-nac

The g-ASCs were marked with QDots-655 (Invitrogen, Life Technologies, USA), for tissue screening *ex vivo*. When they reached 80% of confluence, in *in vitro* cultivation, the g-ASCs were trypsinized, and centrifuged at 462g for 10 min. A total of 3.5 × 10^6^ cells were marked with QDots ^^®^^—emission (655 nm) and excitation (405–615 nm). For marking, the components A and B of the Qdots were homogenised and added to the g-ASCs, they were incubated in an incubator of CO_2_ to 37°C for 50 minutes and stirred every 10 min. It was added 1 mL of half Alpha-MEM (Gibco^™^ Minimum Essential Medium Eagle. Invitrogen, California, USA), analyzed the viability with Trypan Blue 0.4% (Sigma-Aldrich, USA), the cells were counted and centrifuged twice, 462g for five minutes, the *pellet* was resuspended in 1 mL of saline solution and placed in a syringe (1 mL) and an injection was performed in five areas (0.2 mL) directly at the left mammary gland of goats (24h fasting), pre-medicated (0.05 mg/kg xylazine hydrochloride, intramuscular via, after 15 minutes anaesthetised with an association of 8 mg/kg of ketamine and 0.5 mg/kg of maleate midazolam intravenously). The right mammary gland was injected with saline phosphate buffer (PBS Gibco^^®^^, Invitrogen, California, USA) as control. The total number of cells injected in each animal was 3.5 x 10^6^.

For tracking the ASCs, 30 days after cell injection, tissue was collected for biopsy from the right and left mammary glands (lateral and medial region), the goats were anaesthetised with the same protocol described before. It was collected tissue for inclusion in paraffin and for freezing in liquid nitrogen. Histological analysis of paraffined tissues without coloring was performed under a fluorescence microscope (BX41-OLYMPUS) to trace the cell marking with Qdots, and the tissues colored with Haematoxylin-Eosin and Masson’s Trichrome under light microscope (Nikon Eclipse E 200 ^®^, Japan) to describe the structure of the mammary tissue after 30 days of cell injection.

Frozen tissues without coloring, were both analyzed after 90 days of freezing in a fluorescence microscope (BX41-OLYMPUS).

### Evaluation of animals after cell therapy with g-ASCs-nac

After 30 days of cell therapy, sonographic examinations were repeated, and a new collection of the mammary glands tissue was performed, following the same procedures described before the cell therapy.

In the first lactation period after injection of cells, it was performed a physicochemical analysis of the milk by the same ultrasonic method already described (Ekomilk Total ^®^ ultrasonic milk testing device. Eon Trading LLC, Bulgaria).

For analysis of the physicochemical results three groups were formed: animals clinically healthy, control (CTR), animals with chronic mastitis and without treatment with ASCs (M-ASC) and animals with chronic mastitis treated with ASCs (M+ASC).

### Statistical analysis

Statistical data for histological semi quantitative analysis were made by comparison of variable fibrosis, inflammatory of infiltrative cells and cell proliferation between the pre and post injection stage of ASC in goat's mammary gland using Chi-Square test.

In the physico-chemical results, analysis of variance (ANOVA) were performed, aiming to identify statistically significant differences between the independent groups, followed by the comparison multiple Tukey's test, when a treatment effect was detected. A rejection level of the nullity hypothesis of 5% (P ≤ 0.05) was adopted. Statistical analyses were performed using SAS software (Statistical Analysis System, V. 9.3).

## Results

**Fig 1:**
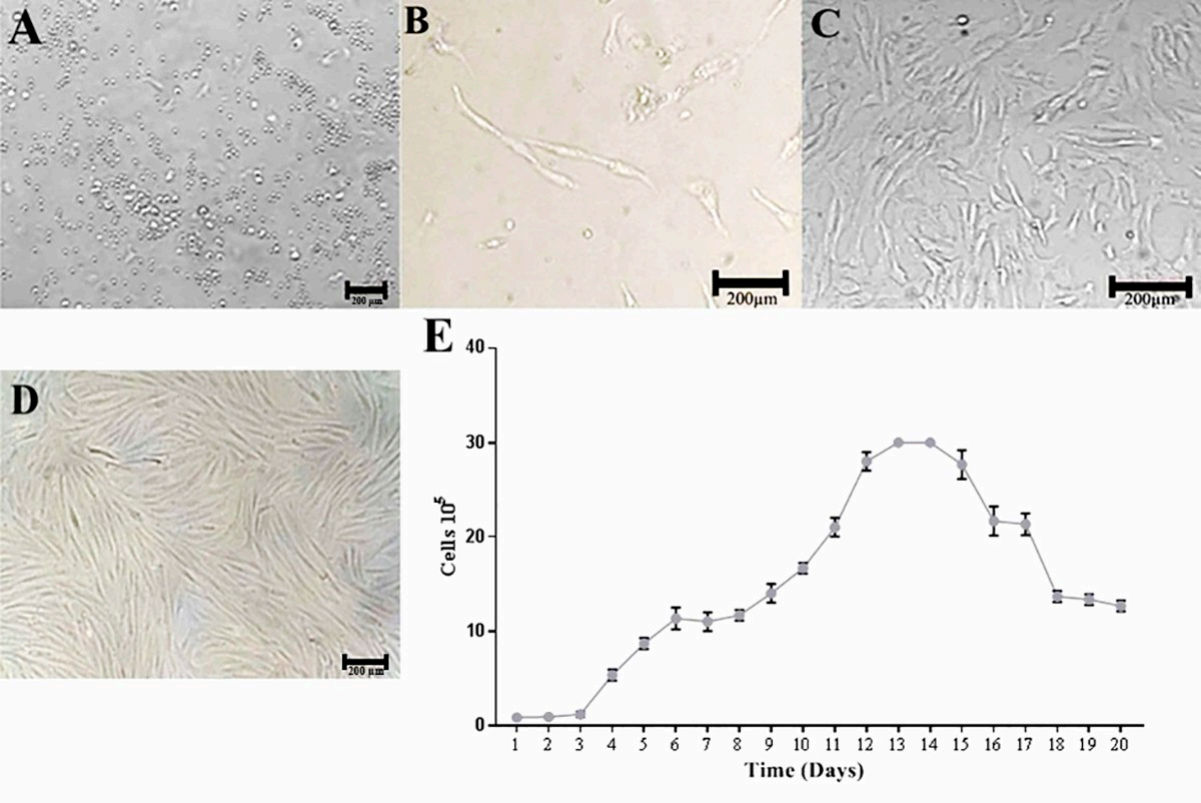
Photomicrography of g-ASCs cell culture isolated from subcutaneous adipose tissue of goats. (A) Stromal fraction of adipose tissue in culture medium DMEM-F12, 24 hours of insulation, 10×10. (B) Cells with fibroblastoid plastic-adherent morphology, five days, 10×10. (C) Cells in 20 days of culture, 80% confluence, 10×10. (D) Cell culture of g-ASC, after four days of thawing, 90% confluence, 10×10. (E) Growth curve (20 days) of in vitro cultured g-ASCs.

In vitro culture, g-ASCs demonstrated high cellularity in 24 hours and viability above 95% (Fig 1A). In five days, isolated cells with fusiform morphology were observed, forming a monolayer of adherent cells (Fig 1B).

DMEM-F12 medium promoted rapid cell multiplication. In 20 days, the culture acquired 80% confluence and characteristic fibroblastoid appearance. The expansion of the culture reaching 80% allowed homogeneity of the cell population and maintenance of indifferentiation (Fig 1C). After cryopreservation, g-ASCs maintained their viability, fully confluent with five days of culture (Fig 1D).

Analyzing the growth curve of the g-ASCs it showed they have rapid proliferation, and it presented a very short lag phase, three days. The log phase started on the fourth day of cultivation. From the ninth day the cell proliferation increased in geometric speed, characterizing successive mitotic divisions. The stability phase, plateau, was observed on the thirteenth day and fourteenth, declining continuously in the following days. After reaching confluence the cells underwent growth inhibition (Fig 1E).

**Fig 2:**
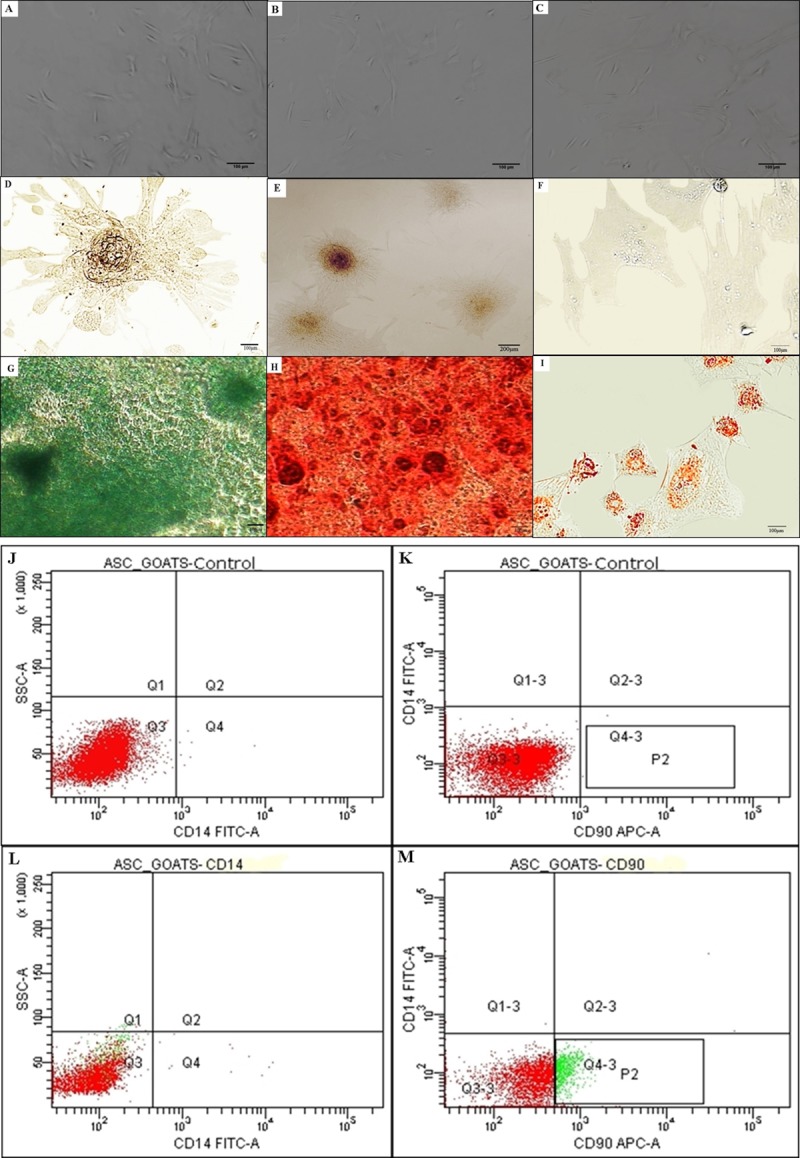
*In vitro* differentiation tests and phenotypic profiles of g-ASCs by flow cytometry. (A, B, C) Differentiation controls: chondrogenic, osteogenic and adipogency, respectively. (D) Suggestive aggregate of chondroblastic cells with formation of spheroid bodies, and deposition of cartilaginous matrix, 10×20. (E) Osteoblasts around calcified bone matrix stained with Alizarin red 10×40. (F) Adipose cells, with intracellular deposit of lipid stained by Oil Red O, 10×20. (G, H, I, J) Cell markers expression analysis by cytometry flow rate. (G, H) Fluorochromes Control CD14, FITC e CD90, APC. (I, J) CD14, FITC e CD90, APC.

The g-ASCs demonstrated the ability to differentiate into other mesenchymal cell lines, proving their plasticity. The chondrogenic differentiation showed that the g-ASC increased in volume and were added, forming a well-defined cell mass, presenting as typical spheroid bodies and beginning to synthesize cartilaginous matrix (Fig 2D).

The osteogenic differentiation showed nodule-like cell aggregates, forming a colony of cells around the calcified bone matrix. The extracellular matrix was stained with Alizarin Red, confirming the differentiation of g-ASCs into cells of the osteogenic lineage (Fig 2E). This way, g-ASCs, when stimulated in vitro, underwent morphological and functional changes to give rise to other types of mature cells. The adipogenic differentiation was characterized by the presence of flattened morphologycal cells and was confirmed by the labeling with Oil Red O, because after 14 days of culture with the adipogenic inducing medium, fat droplets were observed in the cytoplasm of the cells and at 21 days there were differentiation into adipose cells (Fig 2F).

Regarding the phenotypic profile of g-ASCs by flow cytometry, the results demonstrated heterogeneity of the culture, about 30% of the cells expressed CD90, indicator of mesenchymal cells. As for CD14, as expected, there was no marking, excluding the possibility of hematopoietic cells (Fig 2I and 2J).

For the characterization of mastitis, the results of bacterial isolation (Table 2) showed that the isolated microorganism with the highest prevalence was the genre *Staphylococcus*, it was also found fungi of the type *Candida Sp*. The infections were bilateral.

**Table 2:** Microbiological diagnosis of milk with CMT positive mastitis and antibiogram result.

Microbiological Isolation	Antibiogram		
	S	Ms	R
*Staphylococcus* Coagulase Negative	AMI, CIP, IPM, NOR	TRI, GEN	AMO, AMP, AZT, AZI, BAC, CLA, Clo, ERI, ENO, LIN, Oxa, PEN, TET, VAN
*Staphylococcus* Coagulase Positive	AMI, CIP, ENO, GEN, IPM	NOR	AMO, AMP, AZT, AZI, BAC, CLA, Clo, ERI, LIN, Oxa, PEN, TET, TRI, VAN
*Candida Sp+*	AZI, CIP, IPM	GEN, NOR, TRI, TET	AMI, AMO, AMP, AZT, BAC, CLA, Clo, ERI, ENO, LIN, Oxa, PEN, VAN
*Corynebacterium Sp*	AMI, AZI, CIP, Clo, ENO, GEN	NOR	AMO, AMP, AZT, BAC, CLA, ERI, IPM, LIN, Oxa, PEN, TET, TRI, VAN
*Micrococcus Sp*	AMI, AMO, AZI, BAC, CLA, CIP, ENO, GEN, IPM, LIN, TET	Oxa, VAN	AMP, AZT, Clo, ERI, NOR, PEN, TRI

N = 16 milk samples. Animal numbers = 8. Antibiotics: AMI Amikacin; Amoxacyllin; AMP Ampicillin; AZT Azthreomycin; AZI Azithromycin; BAC Bacitracin; CLA Clarithromycin; CIP Ciprofloxacin; CLO Chloramphenicol; ENO Enrofloxacin; ERI Erithromycin; GEN Gentamycin; IPM ImiPenem; LIN Lincomycin; NOR Norfloxacin; OXA Oxacillin; PEN Penicillin G TET Tetracycline. TRI Trimethropin; VAN Vancomycin. Antibiogram: sensitive (S), moderately sensitive (MS), resistant (R).

The SCC of goats showed concordance with microbial isolation, presenting values above 10^6^ in all samples.

**Fig 3:**
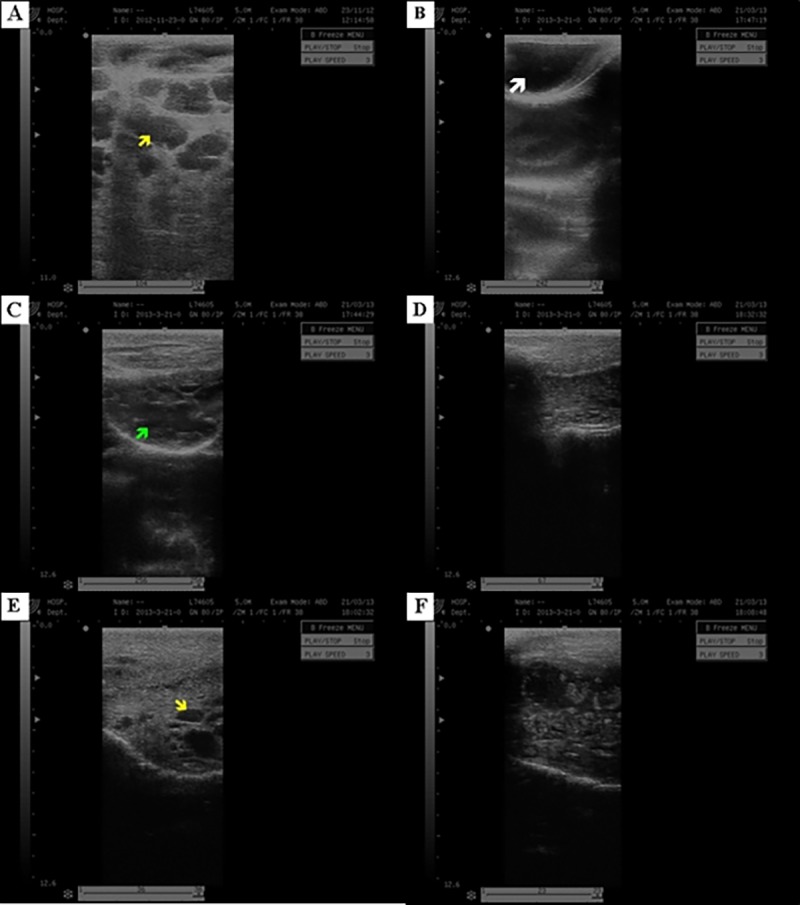
Ultrasonographic image of mammary gland of goats. (A, B) (Group CTR) Gland in the lactation phase, upper and lower, arrows (mammary alveolus–white arrow, cistern- yellow arrow). (C, D) (Group M-ASC) Mammary gland with chronical mastites, involuted alveolus (green arrow). (E, F) (Group M+ASC) Mammary gland after transplanto f ASC, beginning of lactation, with increasing of mammary alveolus.

By ultrasonography, a difference was observed between the echogenicity of the healthy mammary tissue (Fig 3A and 3B) compared to the tissue with chronic mastitis (Fig 3C and 3D). This heterogeneous ecotexture of the mammary tissue was verified in all animals with mastitis. The inflammation caused alterations in the echogenicity of the organs and there was involution of mammary alveoli (Fig 3C and 3D). At the beginning of the lactation period, there were mammary alveoli in formation after g-ASC transplantation (Fig 3E and 3F).

The physicochemical parameters of the milk represented in Table 3 show that there was a significant difference among the groups (CTR, M-ASC and M + ASC) for milk solid-not-fat in the right and left glands; and also fat and temperature in the left gland (S1–S7 Tables).

**Table 3:** Averages, standard deviations and variance analysis of the variables measured in the milk of the right and left mammary gland in the three groups (CTR, M-ASC and M + ASC).

Parameter	CTR	M-ASC	M + ASC	Sig	CTR	M-ASC	M + ASC	Sig
	Right	Right	Right	Right	Left	Left	Left	Left
**Fat** %	4,80±0,68	4,80±1,98	3,30±1,12	ns	4,67±0,74 ^a^	5,43±1,39^a^^b^	3,24±1,01^a^^c^	s
**MSNF** %	7,87±0,79	8,66±0,74	7,79± 0,57	s	7.62±0.46	8,40±0,88	7,81± 0,57	ns
**Den** g/Ml	28,10±3,58	30,65± 1,97	29,16±2,92	ns	27,65±2,33	29,81±3,28	29,48±2,83	ns
**Pro** %	2,69 ± 0,33	3,00± 0,29	2,69 ± 0,24	ns	2,58 ± 0,19 ^a^	2,91± 0,33 ^b^	2,68± 0,26 ^a^	ns
**T** º C	29,30± .08	29,70±0,15	29,80± 0,47	ns	28,65±1,13^a^	29,70± 0,24^b^^c^	29,89±0,50^b^^c^	s
**Lac** %	4,58 ± 0,48	5,01 ± 0,39	4,54 ± 0,39	ns	4,44 ± 0,30	4,87 ± 0,48	4,57 ± 0,38	ns
**Z** Ms/cm	5,12 ± 0,39	5,22 ± 0,24	5,35 ± 0,72	ns	5,25 ± 0,37	5,22 ± 0,24	5,75 ± 0,86	ns
**pH**	7,09 ± 0,08	7,22 ± 0,3	7,06 ± 0,12	ns	7,08 ± 0,09 ^a^	7,24 ± 0,43 ^b^^c^	7,06 ± 0,13 ^b^^c^	ns

Fat; MSNF—milk solid-not-fat; Den-Density; Pro-Protein; Lac-Lactose Z-Electric Conductivity; pH

ns–Averages that do not differ significantly in the milk of the right and left gland

s—scores that differ significantly in the milk of the right and left gland

a, b, c—scores followed by different letters differ significantly by multiple comparison (test of Tukey 5%) among the statistically different groups (CTR, M-ASC and M + ASC) in the right and left glands.

The parameters were compared among groups using the Tukey test and showed that in the left gland, the fat difference occurred among animals from M-ASC and M + ASC, there was also a difference in temperature among the three experimental groups.

**Fig 4:**
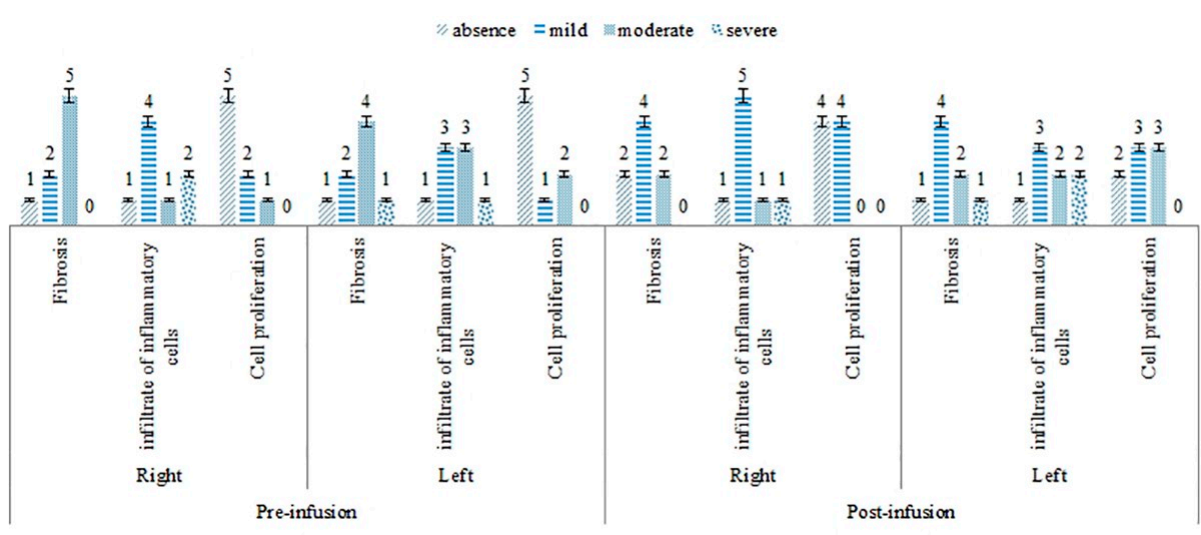
Pre- injection histopathologic evaluation and post- injection with ASCs in the right and left mammary glands of goats with chronic mastitis, based on the parameters fibrosis, infiltrate of inflammatory cells and cell proliferation. (S8–S12 Tables).

In the histopathological evaluation there were alterations in the mammary parenchyma of the animals clinically affected by mastitis before and after cell therapy, being the main lesions the infiltration of inflammatory cells and fibrosis, in which, a small reduction was observed after the application of ASC, although there was no statistically significant difference. The semiquantitative analysis of histopathological slides also demonstrates reduction of fibrosis and inflammatory intensity and increased cell proliferation (Fig 4).

**Fig 5:**
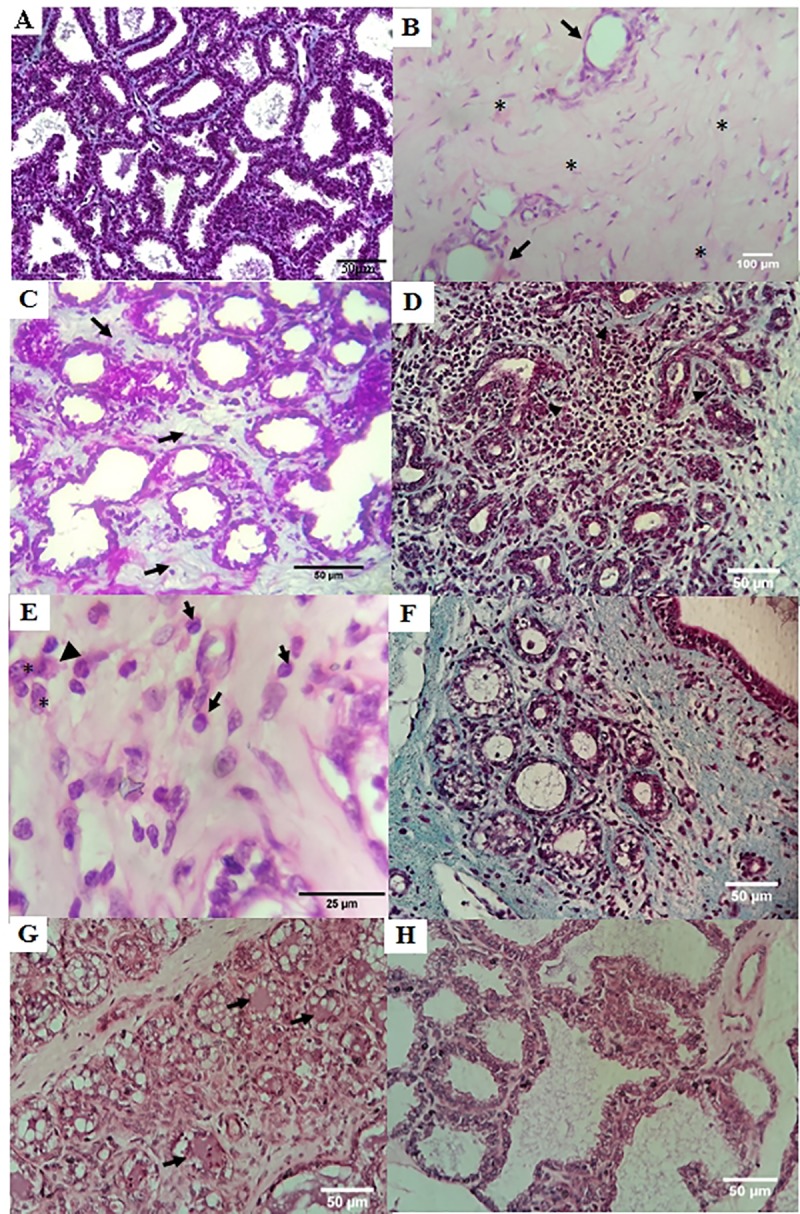
Parenchyma of goat’s mammary gland. (A) Control, regular gland in lactation, TM (B) Chronical mastitis, pre treatment with ASC: Involuted mammary gland, connective tissue (asterisk), excretory duct (arrow) H-E. (C) Intralobular fibrosis (arrow). (D) infiltrate of mixed moderate inflammatory cells, with predominance of lymphocytes, intralobular fibrosis and disorganization of the acinar pattern (arrow head) TM. (E) pattern. chronic mastitis, post-treatment with ASCs: mild, mixed interstitial inflammation consisting of lymphocytes (arrow), plasmocytes (arrow head) and macrophages (asterisk), H-E. (F) Same animal from D, reduction of inflammation and intralobular fibrosis, accini with regular-contour accini, TM. (G) Intralobular ducts with secretion in the lumen (arrow head). (H) normal secretory alveoli in lactating gland, H-E.

Prior to therapy with ASCs the alterations consisted on samples with gland in involution process, with abundance of connective tissue and reduction of epithelial and alveolar areas (Fig 5B), though the animals lactation phase. Fibrosis was characterized by an abundance of interlobular and intralobular collagen fibers (Fig 5C). The interstitial infiltration of inflammatory cells was mixed, varying from mild to severe intensity, consisting of lymphocytes, plasmocytes, macrophages and rare neutrophils (Fig 5D and 5E). In the samples with severe interstitial inflammation, the predominant cell type was lymphocyte (Fig 5E). In the lumen of some accinos and ducts there were neutrophils and epithelium desquamation cells. Degeneration of the acinar epithelium was also evidenced.

After cell therapy with G-Ascs, the same lesions were visualized, however, in a general way in lower intensity. There was a reduction in fibrosis, evidencing mild intensity, with little intralobular connective tissue, accinos with regular contour and evident reduction of inflammatory infiltrative cells (Fig 5F). Samples were observed with intralobular ducts with secretion in the lumen, as well as proliferation of acinar and tubular epithelial cells, which varied from mild to moderate (Fig 5G) and samples of lactating glands with well-developed accinos (Fig 5H) besides the proliferation of acinar and tubular epithelial cells, which varied from mild to moderate.

**Fig 6:**
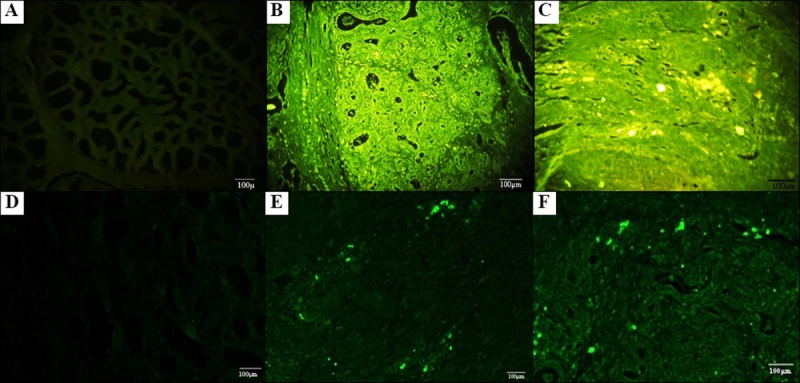
Photomicrography of the mammary gland of goats without staining after 30 days of therapy with g-ASCs. (A) Parafinated tissue without marking with Qdot (control), 10 × 20. (B, C) Parafinate tissue of the right mammary gland (without g-ASCS injection), and left (with g-ASCS injection), respectively, 10x20. (D) Frozen tissue without marking with Qdot (control), 10 × 20. (E, F) Frozen tissue of the right mammary gland (without g-ASCS injection), and left (with g-ASCs injection), respectively, 10x20.

The g-ASCs were labeled with Qdots and after 30 days (Fig 6) of cellular injection in the mammary gland they were screened. Fluorescent signals were visualized on tissue samples ex vivo paraffin embedded (Fig 6B and 6C) and frozen (Fig 6E and 6F) under fluorescence microscopy (BX41-OLYMPUS). The g-ASCs were injected in the left mammary gland, however, the fluorescent signs of Q-Dots were visualized in the two glands confirming the contralateral cell migration. The nanocrystalline particles remained bright in the tissue in analyzes performed on slides with 30, 60 and 90 days of preparation.

## Discussion

The adipose tissue proved to be an important source of stromal cells, low invasive collecting with a large amount of cells. These cells were initially obtained by Zuk et al. [29], and have favorable characteristics for cell therapy [12, 14, 15, 2, 8, 10, 11]. These advantages were described in the isolation, expansion and characterization of ASCs from equines, humans and rats [6, 13, 30, 8].

In the first two days of culture, the characteristic heterogeneity of the primary culture was observed. From the tenth day, the culture became more homogeneous with predominance of the adherent cells, forming a monolayer with elongated fusiform morphology. When the culture reached 100% of confluence, at that moment, due to the high cellularity with density saturation, the culture reached the limit, even the media being changed to nutrient supply, space is a limiting factor, which can lead to cell death. According to Dominici et al [31] this is the first criterion for the characterization of mesenchymal stem cells. Carvalho et al. obtained the same results in horses [32].

The ASCs presented differentiation capacity in osteogenic, chondrogenic, and adipogenic lines, being another important requirement for their characterization according to Dominici et al. [31].

Absence of markup for CD14, is found when the evaluated cells are mesenchymal, because these markers are specific to hematopoietic cells [33]. In terms of expression of specific markers for mesenchymal cells such as CD90, the cell population expressed 30% of surface markers, the results showed heterogeneity of the population.

As to ultrasound images of the mammary gland before cell therapy, there were changes in echogenicity related to mastitis, showing a difference between healthy mammary tissue and with chronic mastitis, mainly regarding the predominance of fibrous solid component.

Research on animal species with mastitis have identified structures with different echogenicities, including fibrosis, intraluminal obstructions and atresia, and can characterize mastitis [34]. Feliciano et al. [4] were also able to visualize alterations in the mammary gland of small animals, through inflammation, through ultrasonographic images.

After the therapy, alterations were observed regarding the tissue organization of the mammary gland, these refer to the beginning of development of the mammary alveoli and presence of fluid.

In the left mammary gland the physical-chemical results of the fat showed differences between the groups (M-ASC and M+ASC) and at the temperature parameter among the three groups evaluated. This allows us to infer that there was progress in terms of functionality of the gland (M+ASC), presenting an amount of fat within the standard [35], in animals with chronic mastitis there may be an increase in the average concentration of fat in the milk. Similar results with increased fatty acids during infectious processes of mastitis were observed by Ogola et al.[36]. According to Leitner et al [37] these higher percentages of fat in infected animals is attributed to the reduction of milk volume.

An important aspect that certifies the progress in the functionality of the gland, is that the injection of the cells was performed in the left gland, and although migration occurred to the right, it was observed by tracing the marked cells with Qdots, it is assumed that the pool of g-ASCs in the left gland is greater than the right, so better response to therapy at the local injection. It is possible to emphasize that fat is the most important component of milk, but also, which presents greater variability [36].

The parameter MSNF showed a significant result, in the right gland, this parameter comprises all the components of the milk, except fat, in this way, although proteins and lactose did not indicate statistical difference, the result of the addition of these components was significant. Zafalon et al. [38] pointed out that changes in this parameter are due to variations in lactose and milk protein content.

Better results could have been obtained in the evaluation of the physicochemical quality of milk if the period of analysis after therapy had been longer, and thus to verify greater functional alterations of the mammary gland.

The histopathological, ultrasonographic, verification of the physicochemical parameters of milk results, as well as considering the return of the gland functionality of the animal’s mammary gland, they show an important biological result in the pre-clinical study of therapy with mesenchymal cells. The therapeutic potential of these cells has been amply demonstrated in experimental studies on muscle atrophy regeneration [39], myocardial ischemia [40], liver disease [1], liver cirrhosis [41], regeneration of periodontal tissue [42], corneal lesions [43], ischemia [44] among others, with varying efficacy.

According to histopathologic findings, the mammary tissue was injected with inflammatory cells, both in the acini and interstitial tissue and proliferation of fibrous connective tissue. This is usually observed in chronic mastitis, causing degeneration of the mammary epithelium [45].

According to Zhao and Lacasse [46] alterations in the mammary tissue are initially caused by microorganisms. Bacteria produces toxins that destroy cell membranes, causing damage to the milk production tissue, while others may invade and multiply within the epithelium before causing cell death, and in these circumstances, mastitis is characterized by the influx of somatic cells. Under these conditions, the acini and affected ducts begin to involute and cease the secretion of milk [47].

Fibrosis is initiated by the response to chemotactic factors released by the damaged cells themselves, so the surrounding connective tissue becomes swollen, then increases in quantity and deposits in the periglandular and periductal region, obliterating lumen, acini and even the cistern, in this way the vascularization of the mammary gland with advancement of the fibrous tissue is compromised, an aspect that contributes to the atrophy and gland malfunction [48].

After cell therapy, inflammatory infiltrate cells was still observed, however some aspects should be considered in order to understand the results. The 30-day period, after therapy, for observation of morphological change in the mammary tissue, may not have been enough to show a greater evolution in the process of reconstitution of the gland.

Barreira et al. [49], when evaluating histologically, equine tendinitis lesions, treated with mesenchymal stem cells 48 days after induction, found reduction of inflammation, with cell proliferation, but there was a need to increase the evaluation period after therapy.

Thus, corroborating the results of Barreira et al [49], it is considered that the process of reorganization of the mammary tissue was not completely finished, after 30 days, but these results are satisfactory, with perspectives of the use of ASCs in therapy. Beheregaray et al [50], also evaluated the activity of mesenchymal stem cells applied to the inflammatory and proliferative phases of wound healing in mice for seven days, the use of ASCs increased vascularization, formation of granulation tissue, collagen deposition and increases the number of hair follicles in just seven days of evaluation, the time of application of the cells did not affect significant differences in the inflammatory and the proliferative phase of wound healing skin.

The intensity of epithelial cell proliferation in the mammary tissue associated with good animal health conditions with characteristic mammary morphology after therapy, are important indicative aspects of potential tissue restoration. According to Blanpain, Horsley, Fuchs[51] naturally most epithelial tissues renew themselves throughout adulthood due to the presence of multipotent stem cells and with ASC injection the results are promising, as for tissue renewal and reconstitution.

Based on the scores used to analyze the characteristics of fibrosis, inflammatory infiltrate cells and cell proliferation, changes were observed before and after therapy. Considering a more descriptive analysis, it was observed that after therapy, there was a tendency to decrease fibrosis intensity, and an increase in cellular proliferation, characterizing neoformation process of resident tissue (immature mammary tissue) developed by stem cells, not observed prior to cell therapy.

Evaluation by cell proliferation scores was related to the intensity of alveolar formation and the organization of the mammary tissue.

Injected stem cells release growth factors and have immunomodulatory function that stimulate resident cells to progress in the cell cycle, from G0 to G1 and from this to S, leading to cell proliferation [15, 16]. In this way, the intervention with injection of stem cells, in chronic inflammation, it is important to contain tissue damage, generated by the replacement of the parenchyma by fibrous tissue.

The marking of g-ASCs with semiconductor fluorescent nanocrystals (Qdots) to screening in the clinical trial of therapy, made possible to overcome the problems associated with conventional coloring, and presents the chance to inject these nanoparticles directly inside living cells. Oliveira et al [52] and Rosen et al. [53] used Qdots for cell marking and tracking satisfactorily. It seems suitable for visualization of stem cells injected into living tissues thanks to their photostability and marking longevity, both in paraffined and frozen tissues. The marked g-ASCs injected in the left mammary gland of goats migrated to the right, these were seen in a different way, with intense brightness, in both glands. The marking with Qdot is stable, this way the marking is not incorporated by residentes tissue cells.

## Conclusion

The treatment of goats with chronic mastitis with adipo derivated stem cells restored the milk production with physicochemical properties appopriate for human consumption. The transplantation of adipose tissue stem cells for the repair of mammary tissue with chronic mastitis is feasible, due to the physiological results obtained, besides being potentially safe for animals, without rejection and since it is a little invasive technique.

The results open new perspectives for the treatment of chronic diseases in the mammary gland of dairy animals, considering the milk production recovery and possible regenerative potential of stem cells from adipose tissue. However, further studies are needed to better establish the protocol for future clinical applications, and to clarify all mechanisms for better post-therapy results.

The accurate identification of subpopulations of ASCs involved in the repair process is another important question to be investigated, in order to know more about the biology of these cells and its therapeutic application.

## Supporting information

S1 File Animal Experimentation Ethics Committee of the Federal University of Piauí. (PDF)

S2 File Pre-injection and post-injection milk production. (PDF)

S1 Table ANOVA test results among variables (Fat, MSNF, Den, Pro, PC, T, Lac, Z, PH, AAL) Right. (PDF)

S2 Table ANOVA test results among variables (Fat, MSNF, Den, Pro, PC, T, Lac, Z, PH, AAL) Left. (PDF)

S3 Table Multiple comparison (Tukey test) of the width variable among the groups, that was statistically different among the means–Right.
(PDF)

S4 Table Multiple comparison (Tukey test) of the width variable among the groups, that was statistically different among the means–Left.
(PDF)

S5 Table Means and standard deviations of variables measured in the right ureter study with 3 groups (control, mastitis without treatment, mastitis with treatment). (PDF)

S6 Table Mean and standard deviations of variables measured in the study of the left subgroup with 3 groups (control, mastitis without treatment, mastitis with treatment). (PDF)

S7 Table Raw data. Mean and standard deviations of variables measured in the study of the left subgroup with 3 groups (control, mastitis without treatment, mastitis with treatment).
(PDF)

S8 Table Original quantitative data from the g-ASC pre-injection histopathology in goat's right mammary glands. (PDF)

S9 Table Original quantitative data from the g-ASC pre- injection histopathology in goat's left mammary glands. (PDF)

S10 Table Original quantitative data from the g-ASC post- injection histopathology in goat's right mammary glands. (PDF)

S11 Table Original quantitative data from the g-ASC post- injection histopathology in goat's left mammary glands. (PDF)

S12 Table Statistical data of the comparison between the variables fibrosis, inflammatory infiltrative and cell proliferation between the pre and post injection stages of g-ASC in the goat's mammary gland. (PDF)
