# Supplementary material for: Adipose stem cells in reparative goat mastitis mammary gland
Source: PLoS One. 2019 Oct 22;14(10):e0223751. doi: 10.1371/journal.pone.0223751 (PMC6804991; doi:10.1371/journal.pone.0223751)
Supplement: S1 File — (PDF) [file pone.0223751.s001.pdf]

S1 File. Animal Experimentation Ethics Committee of the Federal University of Piauí

MINISTÉRIO DA EDUCAÇÃO
UNIVERSIDADE FEDERAL DO PIAUÍ
PRÓ-REITORIA DE PESQUISA E PÓS-GRADUAÇÃO
COMITÊ DE ÉTICA EM EXPERIMENTAÇÃO COM ANIMAIS
Campus Universitário Ministro Petrônio Portela, Bairro Ininga, Teresina, Piauí, Brasil; CEP: 64049-550
Telefone (86) 32155734 – e-mail: ceeapi@ufpi.br

Teresina, 19 de junho de 2012.

Ao (A)

Prof (a): Dra. Maria Acelina Martins de Carvalho
Departamento: Morfofisiologia veterinária
Sr. (a) Pesquisador (a),

Declaro para os devidos fins que o projeto intitulado: **"Cultivo celular e desenvolvimento de protocolo pré-clínico de transplante de células-tronco mesenquimais na terapia da mastite caprina."**, foi avaliado pelo Comitê de Ética em Experimentação com Animais – CEEA/UFPI teve parecer **APROVADO sob o nº. 037/12**. Esclarecemos que o mesmo se encontra de acordo com os requisitos exigidos para apreciação de projetos de pesquisa.

Atenciosamente,

Profª. Ivete L. de Mendonça
Comitê de Ética em Experimentação Animal-UFPI
Coordenadora
