# Supplementary material for: Adipose stem cells in reparative goat mastitis mammary gland
Source: PLoS One. 2019 Oct 22;14(10):e0223751. doi: 10.1371/journal.pone.0223751 (PMC6804991; doi:10.1371/journal.pone.0223751)
Supplement: S2 File — (PDF) [file pone.0223751.s002.pdf]

S2 File. Pre-injection and post-injection milk production (Group M+ASC)

Animal	Milking sessions	Pre injection (mL)	Post injection (mL)
1	1	80	800
	2	80	750
	3	70	810
2 (142)	1	85	120
	2	80	250
	3	80	180
3	1	80	100
	2	80	80
	3	85	100
4	1	90	900
	2	90	810
	3	80	820
5	1	85	800
	2	90	850
	3	90	820
6 (127)	1	80	900
	2	75	810
	3	75	850
7(101)	1	80	300
	2	85	120
	3	85	250
**Means**		**80**	**800**
**Standard deviation**		**5,38**	**340,07**
