# Supplementary material for: Adipose stem cells in reparative goat mastitis mammary gland
Source: PLoS One. 2019 Oct 22;14(10):e0223751. doi: 10.1371/journal.pone.0223751 (PMC6804991; doi:10.1371/journal.pone.0223751)
Supplement: S1 Table — (PDF) [file pone.0223751.s003.pdf]

S1 Table - ANOVA test results among variables (Fat, MSNF, Den, Pro, Pc, T, Lac, Z, PH, AAL) Rigth.

Variable	F calculated	RIGTH<br>F(0,05) Critical (2,20)	p	Significance
Fat	3,044	3,49	0,07	Exist Not
MSNF	3,696	3,49	0,043	Exist
Den	1,511	3,49	0,245	Exist Not
Pro	3,123	3,49	0.066	Exist Not
PC	3,675	3,49	0.044	Exist
T	1,179	3,49	0,328	Exist Not
Lac	3,043	3,49	0,07	Exist Not
Z	0,405	3,49	0,672	Exist Not
PH	1,411	3,49	0,267	Exist Not
AAL	10,32	3,49	0,001	Exist
