# Supplementary material for: Adipose stem cells in reparative goat mastitis mammary gland
Source: PLoS One. 2019 Oct 22;14(10):e0223751. doi: 10.1371/journal.pone.0223751 (PMC6804991; doi:10.1371/journal.pone.0223751)
Supplement: S2 Table — (PDF) [file pone.0223751.s004.pdf]

S2 table - ANOVA test results among variables (Fat, MSNF,Den, Pro, Pc, T, Lac, Z, PH, AAL) Left.

		LEFT		
**Variable**	**F Calculated**	**F(0,05) Critical (2,20)**	**p**	**Significance**
Fat	8,238	3,49	0,002	Exist
MSNF	2,831	3,49	0,083	Exist Not
Den	1,214	3,49	0,318	Exist Not
Pro	3,054	3,49	0,07	Exist Not
PC	3,507	3,49	0,049	Exist
T	6,793	3,49	0,006	Exist
Lac	2,345	3,49	0.122	Exist Not
Z	2,175	3,49	0,14	Exist Not
PH	1,05	3,49	0,368	Exist Not
AAL	10,25	3,49	0,001	Exist
