# Supplementary material for: Adipose stem cells in reparative goat mastitis mammary gland
Source: PLoS One. 2019 Oct 22;14(10):e0223751. doi: 10.1371/journal.pone.0223751 (PMC6804991; doi:10.1371/journal.pone.0223751)
Supplement: S3 Table — Multiple comparison (Tukey test) of the width variable among the groups, that was statistically different among the means–Right. (PDF) [file pone.0223751.s005.pdf]

S3 Table. Multiple comparison (Tukey test) of the width variable among the groups, that was statistically different among the means

	RIGTH			
	**Groups**	**q calculated**	**q(0,05); (20;3)**	**Hypotheses H0**
FAT	CTR x (M-ASC)	0,01	3,578	ACCEPTS
	CTR x (M+ASC)	2,95	3,578	ACCEPTS
	M-ASC x (M+ASC)	3,05	3,578	ACCEPTS
MSNF	CTR x (M-ASC)	3,06	3,578	ACCEPTS
	CTR x (M+ASC)	0,31	3,578	REJECTS
	(M-ASC) x (M+ASC)	3,50	3,578	ACCEPTS
Den	CTR x (M-ASC)	2,44	3,578	ACCEPTS
	CTR x (M+ASC)	1,01	3,578	ACCEPTS
	(M-ASC) x (M+ASC)	1,48	3,578	ACCEPTS
Pro	CTR x (M-ASC)	2,99	3,578	ACCEPTS
	CTR x (M+ASC)	0,00	3,578	ACCEPTS
	(M-ASC) x (M+ASC)	3,10	3,578	ACCEPTS
PC	CTR x (M-ASC)	3,55	3,578	ACCEPTS
	CTR x (M+ASC)	0,66	3,578	REJECTS
	(M-ASC) x (M+ASC)	2,99	3,578	ACCEPTS
T	CTR x (M-ASC)	1,66	3,578	ACCEPTS
	CTR x (M+ASC)	2,07	3,578	ACCEPTS
	(M-ASC) x (M+ASC)	0,43	3,578	ACCEPTS
Lac	CTR x (M-ASC)	2,77	3,578	ACCEPTS
	CTR x (M+ASC)	0,26	3,578	ACCEPTS
	(M-ASC) x (M+ASC)	3,14	3,578	ACCEPTS
Z	CTR x (M-ASC)	1,26	3,578	ACCEPTS
	CTR x (M+ASC)	0,55	3,578	ACCEPTS
	(M-ASC) x (M+ASC)	0,74	3,578	ACCEPTS
PH	CTR x (M-ASC)	1,69	3,578	ACCEPTS
	CTR x (M+ASC)	0,40	3,578	ACCEPTS
	(M-ASC) x (M+ASC)	2,17	3,578	ACCEPTS
AAL	CTR x (M-ASC)	6,40	3,578	REJECTS
	CTR x (M+ASC)	3,87	3,578	ACCEPTS
	(M-ASC) x (M+ASC)	2,62	3,578	ACCEPTS
