# Supplementary material for: Adipose stem cells in reparative goat mastitis mammary gland
Source: PLoS One. 2019 Oct 22;14(10):e0223751. doi: 10.1371/journal.pone.0223751 (PMC6804991; doi:10.1371/journal.pone.0223751)
Supplement: S4 Table — Multiple comparison (Tukey test) of the width variable among the groups, that was statistically different among the means–Left. (PDF) [file pone.0223751.s006.pdf]

S4 Table - Multiple comparison (Tukey test) of the width variable among the groups, that was statistically different among the means

	LEFT			
	**Groups**	**q calculated**	**q(0,05); (20;3)**	**Hypotheses H0**
	CTR x (M-ASC)	1,90	3,578	ACCEPTS
FAT	CTR x (M+ASC)	3,57	3,578	ACCEPTS
	(M-ASC) x (M+ASC)	5,66	3,578	REJECTS
	CTR x (M-ASC)	3,20	3,578	ACCEPTS
MSNF	CTR x (M+ASC)	0,78	3,578	ACCEPTS
	(M-ASC) x (M+ASC)	2,51	3,578	ACCEPTS
	CTR x (M-ASC)	2,06	3,578	ACCEPTS
Den	CTR x (M+ASC)	1,75	3,578	ACCEPTS
	(M-ASC) x (M+ASC)	0,33	3,578	ACCEPTS
	CTR x (M-ASC)	3,33	3,578	ACCEPTS
Pro	CTR x (M+ASC)	1,03	3,578	ACCEPTS
	(M-ASC) x (M+ASC)	2,39	3,578	ACCEPTS
	CTR x (M-ASC)	3,60	3,578	REJECTS
PC	CTR x (M+ASC)	1,05	3,578	ACCEPTS
	(M-ASC) x (M+ASC)	2,64	3,578	ACCEPTS
	CTR x (M-ASC)	4,14	3,578	REJECTS
T	CTR x (M+ASC)	4,87	3,578	REJECTS
	(M-ASC) x (M+ASC)	0,75	3,578	ACCEPTS
	CTR x (M-ASC)	2,96	3,578	ACCEPTS
Lac	CTR x (M-ASC)	0,86	3,578	ACCEPTS
	(M-ASC) x (M+ASC)	2,17	3,578	ACCEPTS
	CTR x (M-ASC)	2,42	3,578	ACCEPTS
Z	CTR x (M+ASC)	0,17	3,578	ACCEPTS
	(M-ASC) x (M+ASC)	2,68	3,578	ACCEPTS
	CTR x (M-ASC)	1,62	3,578	ACCEPTS
PH	CTR x (M+ASC)	0,20	3,578	ACCEPTS
	(M-ASC) x (M+ASC)	1,88	3,578	ACCEPTS
	CTR x (M-ASC)	6,58	3,578	REJECTS
AAL	CTR x (M+ASC)	4,14	3,578	REJECTS
	(M-ASC) x (M+ASC)	2,52	3,578	ACCEPTS
