# Supplementary material for: Adipose stem cells in reparative goat mastitis mammary gland
Source: PLoS One. 2019 Oct 22;14(10):e0223751. doi: 10.1371/journal.pone.0223751 (PMC6804991; doi:10.1371/journal.pone.0223751)
Supplement: S5 Table — (PDF) [file pone.0223751.s007.pdf]

S5 Table. Means and standard deviations of variables measured in the right ureter study with 3 groups (control, mastitis without treatment, mastitis with treatment)

	RIGTH																			
	Fat		MSNF		Den		Pro		PC		T		Lac		Z		PH		AAL	
without mastitis (CTR)	4,80	±0,68	7,87	±0,79	28,10	±3,58	2,69	±0,33	49,90	±4,90	29,30	±1,08	4,58	±0,48	5,12	±0,39	7,09	±0,08	2,10	±1,21
animals with chronic mastitis and without treatment with ASCs (M-ASC)	4,80	±1,97	8,66	±0,74	30,65	±1,97	3,00	±0,28	55,25	±2,91	29,70	±0,15	5,01	±0,39	5,35	±0,72	7,22	±0,31	0,00	±0
animals with chronic mastitis treated with ASCs (M+ASC)	3,30	±1,11	7,79	±0,57	29,16	±2,92	2,69	±0,24	50,89	±4,42	29,80	±0,47	4,54	±0,39	5,22	±0,24	7,06	±0,12	0,83	±1,02
