# Supplementary material for: Adipose stem cells in reparative goat mastitis mammary gland
Source: PLoS One. 2019 Oct 22;14(10):e0223751. doi: 10.1371/journal.pone.0223751 (PMC6804991; doi:10.1371/journal.pone.0223751)
Supplement: S6 Table — (PDF) [file pone.0223751.s008.pdf]

S6 Table - Mean and standard deviations of variables measured in the study of the left subgroup with 3 groups (control, mastitis without treatment, mastitis with treatment)

LEFT

	Fat		MSNF		Den		Pro		PC		T		Lac		Z		PH		AAL	
without mastitis (CTR)	4,67	±0,74	7,62	±0,46	27,65	±2,32	2,58	±0,18	49,40	±2,71	28,65	±1,12	4,44	±0,30	5,25	±0,37	7,08	±0,09	2,23	±1,27
animals with chronic mastitis and without treatment with ASCs (M-ASC)	5,43	±1,39	8,40	±0,88	29,81	±3,28	2,91	±0,33	54,55	±4,22	29,70	±0,24	4,87	±0,48	5,75	±0,86	7,24	±0,43	0,00	±0
animals with chronic mastitis treated with ASCs (M+ASC)	3,24	±1,01	7,81	±0,57	29,48	±2,82	2,68	±0,26	50,90	±4,42	29,89	±0,49	4,57	±0,38	5,22	±0,24	7,06	±0,13	0,83	±1,03
