# Supplementary material for: Adipose stem cells in reparative goat mastitis mammary gland
Source: PLoS One. 2019 Oct 22;14(10):e0223751. doi: 10.1371/journal.pone.0223751 (PMC6804991; doi:10.1371/journal.pone.0223751)
Supplement: S7 Table — Mean and standard deviations of variables measured in the study of the left subgroup with 3 groups (control, mastitis without treatment, mastitis with treatment). (PDF) [file pone.0223751.s009.pdf]

S8 Table. Original quantitative data from the g-ASC pre-infusion histopathology in goat's right mammary glands

Animal	Side	Scores											
		Fibrosis				Inflammatory infiltrate				Cell proliferation			
		0	1	2	3	0	1	2	3	0	1	2	3
**1 Pre-infusion**	Right			1			1			1			
**2 Pre-infusion**	Right	1							1		1		
**3 Pre-infusion**	Right		1						1		1		
**4 Pre-infusion**	Right			1		1						1	
**5 Pre-infusion**	Right		1				1			1			
**6 Pre-infusion**	Right			1			1			1			
**7 Pre-infusion**	Right			1				1		1			
**8 Pre-infusion**	Right			1			1			1			
