# Supplementary material for: Adipose stem cells in reparative goat mastitis mammary gland
Source: PLoS One. 2019 Oct 22;14(10):e0223751. doi: 10.1371/journal.pone.0223751 (PMC6804991; doi:10.1371/journal.pone.0223751)
Supplement: S8 Table — (PDF) [file pone.0223751.s010.pdf]

S9 Table. **Original quantitative data from the g-ASC pre-infusion histopathology in goat's left mammary glands**

Animal	Side	Scores											
		Fibrosis				Inflammatoy infiltrate				Cell proliferation			
		0	1	2	3	0	1	2	3	0	1	2	3
1 Pre-infusion	Left	1					1			1			
2 Pre-infusion	Left		1				1			1			
3 Pre-infusion	Left			1					1		1		
4 Pre-infusion	Left			1		1						1	
5 Pre-infusion	Left		1				1			1			
6 Pre-infusion	Left				1			1		1			
7 Pre-infusion	Left			1				1		1			
8 Pre-infusion	Left			1				1				1	
