# Supplementary material for: Adipose stem cells in reparative goat mastitis mammary gland
Source: PLoS One. 2019 Oct 22;14(10):e0223751. doi: 10.1371/journal.pone.0223751 (PMC6804991; doi:10.1371/journal.pone.0223751)
Supplement: S9 Table — (PDF) [file pone.0223751.s011.pdf]

S10 Table. **Original quantitative data from the g-ASC post-infusion histopathology in goat's right mammary glands**

Animal	Side	Scores											
		Fibrosis				Inflammatory infiltrate				Cell proliferation			
		0	1	2	3	0	1	2	3	0	1	2	3
**1 Post-infusion**	right	1				1					1		
**2 Post-infusion**	right			1			1				1		
**3 Post-infusion**	right		1						1		1		
**4 Post-infusion**	right		1				1			1			
**5 Post-infusion**	right	1					1			1			
**6 Post-infusion**	right		1				1			1			
**7 Post-infusion**	right			1				1			1		
**8 Post-infusion**	right		1				1			1			
