# Supplementary material for: Adipose stem cells in reparative goat mastitis mammary gland
Source: PLoS One. 2019 Oct 22;14(10):e0223751. doi: 10.1371/journal.pone.0223751 (PMC6804991; doi:10.1371/journal.pone.0223751)
Supplement: S10 Table — (PDF) [file pone.0223751.s012.pdf]

S11 Table. Original quantitative data from the g-ASC post-infusion histopathology in goat's left mammary glands

Animal	Side	Scores											
		Fibrosis				Inflammatory infiltrate				Cell proliferation			
		0	1	2	3	0	1	2	3	0	1	2	3
**1 Post-infusion**	Left	1				1					1		
**2 Post-infusion**	Left			1					1		1		
**3 Post-infusion**	Left		1						1		1		
**4 Post-infusion**	Left				1		1			1			
**5 Post-infusion**	Left		1				1					1	
**6 Post-infusion**	Left		1				1			1			
**7 Post-infusion**	Left			1				1				1	
**8 Post-infusion**	Left		1					1				1	
