# Supplementary material for: Adipose stem cells in reparative goat mastitis mammary gland
Source: PLoS One. 2019 Oct 22;14(10):e0223751. doi: 10.1371/journal.pone.0223751 (PMC6804991; doi:10.1371/journal.pone.0223751)
Supplement: S11 Table — (PDF) [file pone.0223751.s013.pdf]

S12 Table. **Statistical data of the comparison between the variables fibrosis, inflammatory infiltrative and cell proliferation between the pre and post infusion stages of g-ASC in the goat's mammary gland**

Variable	Right			left		
	**X^2**	**p**	**Result**	**X^2**	**p**	**Result**
**Fibrosis**	2,28	0,32	accepts H0	1,33	0,72	accept H0
**Inflammatory infiltrate**	0,44	0,93	accepts H0	0,53	0,91	accept H0
**Cell proliferation**	1,77	0,41	accepts H0	2,48	0,29	accept H0

Ho - here was no statistically significant difference between the pre-infusion and post-infusion variables for $p < 0.05$
